# Editorial: Lateral ankle sprain, chronic ankle instability and ankle osteoarthritis: unraveling mechanisms and exploring management approaches

**DOI:** 10.3389/fspor.2026.1898800

**Published:** 2026-07-01

**Authors:** Jia Han, Michelle Smith, Phil Newman, Kyeongtak Song, Yinghui Hua

**Affiliations:** 1 Department of Rehabilitation, Jiangwan Hospital, Shanghai University of Medicine and Health Sciences, Shanghai, China; 2 School of Health and Biomedical Sciences, Royal Melbourne Institute of Technology University, Melbourne, VIC, Australia; 3 School of Health and Rehabilitation Sciences, The University of Queensland, Brisbane, QLD, Australia; 4 Centre for Innovation in Pain and Health Research (CIPHeR), The University of Queensland, Brisbane, QLD, Australia; 5 Research Institute for Sport and Exercise (UCRISE), University of Canberra, Canberra, ACT, Australia; 6 Department of Physical Education, Yonsei University, Seoul, Republic of Korea; 7 International Olympic Committee Research Centre Korea, Yonsei University, Seoul, Republic of Korea; 8 Department of Sports Medicine, Huashan Hospital, Fudan University, Shanghai, China

**Keywords:** ankle injuries, biomechanics, clinical assessment, proprioception, rehabilitation

Lateral ankle sprain (LAS) is among the most common musculoskeletal injuries in athletic and general populations (1). Although LAS is often regarded as a minor or self-limiting injury, many individuals experience residual symptoms, recurrent sprains, perceived instability, impaired function, and/or reduced participation after the initial episode (2). These persistent problems may contribute to chronic ankle instability (CAI), a multifactorial condition involving pathomechanical, sensory-perceptual, and motor-behavioral impairments (3). Recurrent instability and altered joint loading may also be linked with cartilage degeneration and post-traumatic ankle osteoarthritis (OA), which can substantially affect pain, mobility, participation, quality of life, and work-related function (4, 5). Therefore, understanding the clinical and mechanistic links from acute sprain through persistent instability to joint degeneration, and identifying stage-appropriate assessment and management strategies, remain questions of considerable clinical and scientific urgency.

This Research Topic brings together 22 contributing articles that address this continuum from complementary perspectives. Collectively, these studies examine mechanisms of instability, movement and loading adaptations, proprioceptive and neural factors, clinical measurement, non-surgical and adjunctive interventions, surgical outcomes, pediatric ankle trauma, and ankle OA prognosis. Rather than viewing LAS, CAI, and ankle OA as isolated clinical entities, the Research Topic highlights the need for multidimensional approaches to reduce recurrent injury, improve function, and protect long-term ankle joint health.

## 1 Biomechanical and structural mechanisms

Unraveling the biomechanical consequences of ankle instability is a central theme of this collection. Wang et al. used dynamic biplanar radiography to show that individuals with CAI demonstrate altered three-dimensional motion of the ankle joint complex during walking, together with changes in vertical ground reaction forces and center-of-pressure trajectories. These findings support the view that CAI involves not only self-reported instability, but also measurable alterations in *in-vivo* joint motion and foot-ground interaction, providing mechanistic insight into how recurrent instability may be associated with altered joint loading patterns relevant to long-term cartilage health.

Furthermore, the biomechanical consequences of ankle instability extend well beyond the ankle joint itself (3). Ma et al. extended this movement perspective to obstacle crossing, showing that individuals with functional ankle instability adopted altered postural-control strategies, including proximal compensation and reduced mediolateral stability during landing. Xu et al. and Cui et al. further demonstrated that sport-relevant tasks, such as side-cutting and single-leg drop landings, may reveal sex-specific lower-limb biomechanical adaptations. These task and sex-specific findings suggest that movement assessment and rehabilitation planning should consider the specific demands of the activity being tested. Similarly, Lin et al. showed that repeated vertical jumping can expose kinetic asymmetry and altered push-off strategies that may not be evident from gross performance outcomes alone. Together, these studies emphasize that LAS-related sequelae should be evaluated under tasks that resemble the physical demands of sport and daily activity.

Structural and computational bioengineering approaches provide mechanistic insights at the tissue and joint-system levels that *in vivo* experimentation alone cannot fully capture, further illuminating the continuum from instability to joint degeneration and surgical prognosis. Ji et al. used finite-element modeling to examine how distinct patterns of injury to the anterior talofibular ligament and calcaneofibular ligament influence ankle stability and talar cartilage stress. Their findings suggest that different ligament components may contribute uniquely to anterior and rotational stability, and that combined ligament injuries may alter cartilage stress distribution in ways that are relevant to post-traumatic joint degeneration. Complementing this mechanistic perspective, Ji and Liu developed machine-learning models that integrated preoperative computed tomography-derived subchondral bone parameters with clinical indicators to predict long-term adverse outcomes after total ankle arthroplasty. They identified subchondral bone mineral density, trabecular separation, and preoperative talar necrosis volume as important predictors. Although these approaches require further validation before routine clinical implementation, they illustrate how bioengineering, imaging, and data-driven modeling can bridge biomechanical understanding with individualized surgical prognosis and long-term ankle OA management.

## 2 Sensorimotor and neural mechanisms

At the neuromuscular level, this collection challenges the view that CAI is primarily a structural ligament problem. Ankle proprioception is important for balance control in sport and may contribute to both performance and lower-limb injury risk (6). However, proprioception should not be treated as a single generic construct, as distinct testing methods may capture different sensory modalities and task-specific abilities (7). Li et al. provided a comparative evaluation of proprioceptive submodalities in individuals with CAI, showing that deficits were not uniform across all sensory domains but were most evident in kinesthesia, force sense, and landing proprioception. This finding is consistent with systematic reviews showing that CAI is associated with proprioceptive deficits, which vary according to movement direction, testing method, and proprioceptive subdomain (8, 9). The task-specific proprioception assessment, the Ankle Inversion Discrimination Apparatus for Landing, is particularly relevant because it assesses proprioception during a functional landing task and may help refine what clinicians measure and target during rehabilitation (10).

To target these sensorimotor deficits, Friedman and Madsen examined the effect of stochastic resonance during gait, suggesting that subthreshold sensory stimulation may influence cutaneous reflex consistency and perceived instability in people with CAI. This work identifies a potentially accessible peripheral neuromodulatory approach for sensorimotor rehabilitation, although its clinical application requires further testing. Moving beyond the periphery, two further studies explored central nervous system (CNS)-targeted modulation. Ge et al. found that high-definition transcranial direct current stimulation combined with Bosu ball training improved selected mediolateral postural-stability outcomes more than Bosu ball training alone. Similarly, Gao et al. showed that transcranial direct current stimulation combined with Bosu ball training reduced ankle inversion and internal rotation during side-cutting, although it did not provide additional performance benefits. This dissociation between biomechanical risk modification and performance enhancement suggests that CNS-targeted approaches may affect specific movement features without necessarily improving gross task performance, highlighting the need for furthe dose-response and implementation research.

## 3 Assessment and clinical decision-making

Accurate assessment is essential for translating mechanistic findings into individualized care. Consensus recommendations for acute LAS emphasize structured clinical assessment, including injury mechanism, ligament and bone assessment, and evaluation of mechanical and sensorimotor impairments (11). Lee et al. examined patients with acute LAS and found that clinical impairments such as swelling and pain were strongly associated with ankle disability, reinforcing the importance of early patient-centered assessment after injury. Clinical practice guidelines also recommend assessing swelling, range of motion, talar translation, talar inversion, balance, and activity limitation across acute LAS, postacute LAS, and CAI care continua (12).

For chronic presentations, Luan et al. highlighted the need for careful selection of patient-reported outcome measures in CAI, emphasizing that instruments should identify instability, detect clinically meaningful symptom change, and remain interpretable across cultural contexts. Standardized CAI selection criteria remain important for research comparability and clinical translation (13). In a separate mini-review, Luan et al. revisited the anterior drawer test and concluded that it remains clinically useful for assessing mechanical ankle instability, including a reported minimal detectable change of 1.995 mm, while also highlighting persistent limitations in inter-rater reliability and the need for standardized testing procedures. Further contributing to clinical utility, Tourillon et al. proposed a practical decision-making framework for restoring ankle dorsiflexion range of motion in athletes using the weight-bearing lunge test, confirmatory assessment, and targeted interventions. Together, these articles show that precision management begins with precision assessment: integrating symptoms, patient-reported outcomes, mechanical stability, range of motion, sensorimotor control, functional performance, and sport-specific demands.

## 4 Conservative, adjunctive, and surgical management

Several articles addressed non-surgical management and prevention, reflecting the field's shift toward multimodal and impairment-based rehabilitation. Previous clinical practice guidelines recommend proprioceptive and neuromuscular therapeutic exercise for individuals with CAI (12). Zhang et al. performed a network meta-analysis on therapeutic exercise for ankle inversion muscle function in individuals with CAI, finding that combined neuromuscular and strength training showed the most favorable comparative ranking among the exercise approaches evaluated. This finding is consistent with previous evidence that individuals with CAI may show strength deficits across the ankle, knee, and hip muscle musculature (14). Furthermore, it suggests that strengthening may be most clinically meaningful when integrated with neuromuscular training rather than used in isolation (15). Whole-body vibration has also been investigated as a sensorimotor intervention for CAI, with evidence suggesting potential benefits for balance, strength, joint position sense, and selected muscle-activity outcomes (16). In this context, He et al. provided randomized trial evidence suggesting that adding whole-body vibration training to strength training and acupuncture may further improve selected outcomes, including ankle function, plantarflexion strength, and anteroposterior balance control, in individuals with CAI.

Regarding adjunctive management, Wu et al. examined the effect of different midfoot wrap pressures in amateur basketball athletes and showed that external support may influence balance and dynamic stability, providing biomechanically grounded findings that may inform sport-specific taping and bracing decisions. Becker et al. found that sensorimotor foot orthoses improved static balance in healthy adults, particularly when visual information was constrained. This observation is clinically relevant because people with CAI may rely more on visual information during postural control (17), suggesting that external sensory input may be especially important when visual cues are limited. Reviewing passive modalities, Chia et al. systematically evaluated pulsed electromagnetic field therapy for foot and ankle soft-tissue pathologies, reporting potential benefits for pain but less consistent evidence for physical function. Together, these studies suggest that non-surgical management after ankle injury should combine exercise, sensory input, external support, adjunctive physical agents, and individualized clinical reasoning according to the patient's impairments, goals, and stage of recovery.

In surgical management, Duan et al. retrospectively examined patients undergoing anterior talofibular ligament reconstruction and found that preoperative anxiety or depressive symptoms were common and associated with worse clinical scores before and after surgery, although improvements in pain and function were comparable between patients with and without these symptoms. This finding highlights the potential value of considering psychological status alongside mechanical instability when planning and evaluating surgical care (18). Zhang et al. used magnetic resonance imaging to characterize periosteal entrapment in pediatric distal tibial Salter-Harris type II fractures, showing that imaging can refine injury morphology and support treatment decision-making without necessarily mandating aggressive operative intervention, as non-surgical management and surgical fixation yielded comparable functional outcomes. At the other end of the continuum, Ji and Liu's aforementioned machine-learning models integrated computed tomography-derived subchondral bone parameters and clinical indicators to predict long-term adverse outcomes after total ankle arthroplasty, illustrating the potential role of imaging-based prognosis in advanced ankle OA. These articles broaden the scope of ankle injury research from recurrent instability alone to psychological health, growth-related injury, imaging-guided decision-making, advanced ankle OA, and long-term joint preservation.

## 5 Synthesis and future directions

The articles complied in this Research Topic reinforce the imperative to view LAS, CAI, and ankle OA as related but heterogeneous clinical problems rather than isolated diagnoses. Across the collection, several cross-cutting messages emerge. First, persistent ankle symptoms are expressed not only through local ligament or joint impairment, but also through altered lower-limb movement strategies, multifaceted proprioceptive deficits, postural-control changes, and patient-reported limitations. Second, task selection matters: walking, obstacle crossing, cutting, landing, jumping, and balance uniquely unmask distinct dimensions of post-injury impairment. Third, clinical management should integrate mechanical assessment, patient-reported outcomes, movement testing, sensory and neuromuscular rehabilitation, external support or adjunctive therapies when appropriate, and consideration of psychological and long-term joint-health factors. Future research should prioritize prospective longitudinal designs, harmonized injury definitions, standardized outcome measures, sex- and task-specific analyses, and trials testing whether mechanism-informed rehabilitation can effectively reduce recurrence and preserve long-term ankle joint health.

Taken together, this collection highlights both the complexity of ankle injury sequelae and the opportunity to develop more targeted, clinically translatable approaches across the LAS–CAI–ankle OA continuum. We thank all contributing authors, reviewers, and editorial staff for making this Research Topic possible, and hope it will serve as a useful resource for future work aimed at improving recovery, reducing recurrence, and preserving long-term ankle joint health.
